# Antigen-Specific CD4^+^ T-Cell Activation in Primary Antibody Deficiency After BNT162b2 mRNA COVID-19 Vaccination

**DOI:** 10.3389/fimmu.2022.827048

**Published:** 2022-02-14

**Authors:** Kai M. T. Sauerwein, Christoph B. Geier, Roman F. Stemberger, Hüseyin Akyaman, Peter Illes, Michael B. Fischer, Martha M. Eibl, Jolan E. Walter, Hermann M. Wolf

**Affiliations:** ^1^ Immunology Outpatient Clinic, Vienna, Austria; ^2^ Department for Biomedical Research, Center of Experimental Medicine, Danube University Krems, Krems an der Donau, Austria; ^3^ Biomedizinische Forschung & Bio-Produkte AG, Vienna, Austria; ^4^ USF Health Department of Pediatrics, Division of Allergy/Immunology, Children´s Research Institute, St. Petersburg, FL, United States; ^5^ Clinic for Blood Group Serology and Transfusion Medicine, Medical University of Vienna, Vienna, Austria; ^6^ Division of Allergy and Immunology, Department of Pediatrics, Morsani College of Medicine, University of South Florida, Tampa, FL, United States; ^7^ Division of Allergy/Immunology, Department of Pediatrics, Johns Hopkins All Children’s Hospital, St. Petersburg, FL, United States; ^8^ Medical School, Sigmund Freud Private University, Vienna, Austria

**Keywords:** circulating follicular T helper cells, CXCR5-negative CD4^+^ memory T cells, common variable immunodeficiency, primary immunoglobulin isotype deficiency, activation induced marker assay, surrogate virus neutralization assay

## Abstract

Previous studies on immune responses following COVID-19 vaccination in patients with common variable immunodeficiency (CVID) were inconclusive with respect to the ability of the patients to produce vaccine-specific IgG antibodies, while patients with milder forms of primary antibody deficiency such as immunoglobulin isotype deficiency or selective antibody deficiency have not been studied at all. In this study we examined antigen-specific activation of CXCR5-positive and CXCR5-negative CD4^+^ memory cells and also isotype-specific and functional antibody responses in patients with CVID as compared to other milder forms of primary antibody deficiency and healthy controls six weeks after the second dose of BNT162b2 vaccine against SARS-CoV-2. Expression of the activation markers CD25 and CD134 was examined by multi-color flow cytometry on CD4^+^ T cell subsets stimulated with SARS-CoV-2 spike peptides, while in parallel IgG and IgA antibodies and surrogate virus neutralization antibodies against SARS-CoV-2 spike protein were measured by ELISA. The results show that in CVID and patients with other milder forms of antibody deficiency normal IgG responses (titers of spike protein-specific IgG three times the detection limit or more) were associated with intact vaccine-specific activation of CXCR5-negative CD4^+^ memory T cells, despite defective activation of circulating T follicular helper cells. In contrast, CVID IgG nonresponders showed defective vaccine-specific and superantigen-induced activation of both CD4^+^T cell subsets. In conclusion, impaired TCR-mediated activation of CXCR5-negative CD4^+^ memory T cells following stimulation with vaccine antigen or superantigen identifies patients with primary antibody deficiency and impaired IgG responses after BNT162b2 vaccination.

## Introduction

COVID-19 (Coronavirus Disease-2019) is caused by infection with SARS-CoV-2, a novel coronavirus discovered at the end of 2019 (1). Interaction between angiotensin converting enzyme 2 (ACE2) highly expressed on human airway epithelial cells and the receptor binding domain of the viral spike protein mediates entry of SARS-CoV-2 into the cell, thereby establishing infection of the host (2). SARS-CoV-2-infected individuals may develop potentially life-threatening pneumonia and respiratory failure in the course of a severe acute respiratory syndrome (SARS) associated with high mortality (3).

Defects of innate and adaptive immunity such as impaired type I interferon response (4), loss of function variants of the X-chromosomal TLR7 gene (5) or predominantly antibody deficiency (PAD) can be responsible for severe COVID-19 with high hospitalization and infection fatality rates (6, 7). Among the PAD group patients with common variable immunodeficiency (CVID) complicated by inflammatory, autoimmune or respiratory comorbidities were most vulnerable to develop severe COVID-19 (6–8). CVID is the most common clinically severe form of primary antibody deficiency, characterized by a severe impairment to produce pathogen-specific IgG antibodies (9). Other forms of PAD (oPAD) show a persistent and marked decrease of at least one of the serum immunoglobulins and/or IgG-subclasses and/or a specific antibody deficiency to polysaccharide antigens but have an intact ability to produce IgG antibodies after vaccination with T-dependent protein antigens (10). Although clinical presentation of these patients is often mild as compared to CVID, severe disease can still develop (11) and these patients can also present with severe COVID-19 (8).

The most effective protection against infection with SARS-CoV-2 is achieved through induction of antibody and T cell responses following vaccination against SARS-CoV-2 spike protein, e.g., with BNT162b2, a new COVID-19 mRNA vaccine (12, 13). As in other viral infections vaccine-induced neutralizing IgG antibody responses are generally considered to be a surrogate marker for immune protection (14, 15). CVID patients might still be susceptible to infection after vaccination as impaired IgG antibody and B cell memory responses have been described following immunization with conventional vaccines, e.g., against seasonal influenza (16, 17) and also with BNT162b2 in one study (18) but not in another (19). The mechanism whereby in a subgroup of CVID patients BNT162b2 vaccination induces IgG antibody formation (18, 19) remains to be studied. In addition, immune responses to BNT162b2 in patients with immunoglobulin isotype deficiency and/or selective antibody deficiency, known to be capable of producing IgG antibodies to conventional vaccines, have not been studied in detail yet.

The generation of B cell memory is critical for the efficacy of a vaccine. CD4^+^ T follicular helper (Tfh) cells promote long-lived humoral immunity after vaccination by providing help to B cells in germinal center reaction in follicles of secondary lymphoid organs (20, 21). Circulating T follicular helper cells (cTfh) contained within the CXCR5^+^ memory CD4^+^ T cell compartment in peripheral blood reflect the Tfh present in germinal center follicles of secondary lymphoid organs (20). In convalescent individuals, SARS-CoV-2-specific cTfh responses correlate with antibody neutralization found within two months following symptoms onset (22), and cTfh responses also participate in IgG antibody production to SARS-CoV-2 spike protein contained in the currently available vaccines (23). In CVID patients divergent results have been reported regarding vaccine-specific T cell responses as identified by IFN-gamma production. While intact T cell responses were found following influenza (24) or BNT162b2 vaccination (19), vaccine-specific T cells did not increase following BNT162b2 immunization in another more recent study (18). CTfh abnormalities have been described in CVID patients (25), but cTfh responses following mRNA vaccination against COVID-19 have not been studied in patients with PAD. In the present study we investigated antigen-specific CD4^+^ T memory subset response using an activation-induced marker assay (26), and formation of IgG and IgA antibodies and also surrogate virus neutralization antibodies against SARS-CoV-2 spike protein by ELISA in patients with PAD vaccinated with two doses of BNT162b2 mRNA vaccine. Our findings indicate that the majority of PAD patients produce normal levels of functional IgG antibody responses following BNT162b2 vaccination and should thus be protected from COVID-19. In addition, abnormalities in vaccine-specific CD4^+^ T cell responses characterize PAD patients with defective IgG responses.

## Patients and Controls

A total of 31 adult patients diagnosed with CVID according to the ESID registry working definitions for the clinical diagnosis of inborn errors of immunity (10) were enrolled in the study (median age in years [IQR] (range): 45 [37–57] (19–85); m/f 12/19) (**Table 1**). All CVID patients received regular SCIG or IVIG substitution therapy and had never experienced a previous SARS-CoV-2 infection as nasopharyngeal swabs were repeatedly negative by PCR testing; the patients lived in social isolation and experienced no clinical symptoms indicative of viral infection. Serum and peripheral blood mononuclear cells were collected 40 days (median, IQR: 34–57) following the second dose of the Pfizer-BioNTech COVID-19 vaccine BNT162b2 (Comirnaty) to determine vaccination responses as part of the routine medical attendance. Monogenetic variants known to be associated with CVID were present in three patients: an IKZF1 mutation in two IgG-responders and an NFKB1 mutation in one IgG-nonresponder; in all other CVID patients the presence of gene mutations associated with other primary immunodeficiency disorders, and also any known monogenetic cause of CVID phenotype was excluded by targeted gene sequence analysis (Illumina technology performed on a MiSeq NGS). Eleven CVID patients without any exposure to SARS-CoV-2 were tested before they received COVID-19 vaccination. For comparison 39 patients with other, milder forms of predominantly antibody deficiency (oPAD) were included in the study (median age in years [IQR] (range): 52 [38–71] (18–90); m/f 13/26), 28 with regular immunoglobulin substitution therapy (fifteen of these had selective antibody deficiency against polysaccharide antigens (SPAD), ten with concomitant IgG subclass deficiency; 13 had IgG subclass deficiency without SPAD), eleven without immunoglobulin replacement (one had selective IgG2-antibody deficiency, six had IgG subclass deficiency, two with concomitant IgA deficiency, four had IgM deficiency). A median of 35 days (IQR 28–59) following the second dose of Pfizer-BioNTech COVID-19 vaccine their immune responses were tested. Eleven oPAD patients without any exposure to SARS-CoV-2 were tested before vaccination. Twenty healthy adults [median age in years (IQR) (range): 62 (47–67) (26–73); m/f 5/15] served as a control group and were tested 39 days (median, IQR: 24–54) following the second dose of Pfizer-BioNTech COVID-19 vaccine; sixteen healthy adults who refused to get a COVID-19 vaccine and were repeatedly tested negative for SARS-CoV-2 infection wanted to know whether they were protected from COVID-19 and served as a SARS-CoV-2-negative healthy control group.

**Table 1:** Characterization of CVID patients with or without IgG-antibody production following second BNT162b2 mRNA COVID-19 vaccination.

		CVID responders (n=15)			CVID nonresponders (n=16)			Normal range
sex (m/f)		4/11			8/8			
		median	IQR		median	IQR		
age at time of diagnosis (yr)		34	26.5	46.4	32	26.6	47.3	
age at time of second BNT162b2 vaccination (yr)		49	36.5	58.5	42	36.5	55	
days between second vaccination and testing of immune response								
		40	35.5	50.5	40	32.8	61	
serum immunoglobulins (mg/dl)								
IgG		363	221.8	558	121	76.5	218	790-1700
IgA		<6	<6	34.5	<6	<6	8	76-450
IgM		37	18.3	60.8	16	<6	63	90-350
serum antibody levels								
23-valent PnP-IgG (reciprocal titer)		<20	<20	<20	<20	<20	<20	428-10785
23-valent PnP-IgM (reciprocal titer)		25	<20	38.5	<20	<20	<20	164-11943
Tet-IgG (IU/ml)		0.13	0.12	0.35	0.09	0.04	0.24	1.67-12.14
Di-IgG (IU/ml)		0.04	0.01	0.19	0.01	0.005	0.02	0.42-7.22
Hib-IgG (ug/ml)		0.15	0.11	0.29	0.11	0.06	0.45	>1
Lymphocyte subpopulations								
CD19 % of lymphocytes		13	10.64	15	7	5.07	13.3	7-23
CD19 abs. number/ul		164	142.75	271.5	88	53.5	250	71-549
CD4 % of lymphocytes		39	33.8	43.25	40	32.5	45	31-66
CD4 abs. number/ul		457	377	579	640	509.75	708.5	386-2022
CD8 % of lymphocytes		36	26.6	36.7	38	31.27	52	21-43
CD8 abs number/ul		342	253	564	547	436	1192	297-1011
B cell subpopulations (% of CD19-positive lymphocytes)								
IgD+CD27-		73	65.37	85.38	86	77.9	91.3	45.84-80.36
IgD+CD27+		23	10.46	29.42	8	4.29	16.9	6.81-30.53
IgD-CD27+		3	2.01	5.05	2	0.76	2.28	7.81-27.45
Lymphocyte proliferation (dpm)								
PHA		98790	62713	162669	92454	582373	131827	>20000

## Materials and Methods

### Measurement of Antibody Responses After BNT162b2 Vaccination

Antibody response was evaluated by testing IgG- and IgA-antibodies against the recombinant S1 domain of SARS-CoV-2 spike (S) protein (containing the receptor-binding domain) with commercially available quantitative [anti-SARS-CoV-2-Quantivac-ELISA (IgG), Euroimmun Medizinische Labordiagnostika AG, 23560 Lübeck, Germany] and semiquantitative [anti-SARS-CoV-2-ELISA (IgA), Euroimmun Medizinische Labordiagnostika AG] ELISA kits used according to the manufacturer’s instructions. Results for IgG antibodies are expressed as relative units (RE)/ml, values ≥11 RE/ml are considered positive. These relative units can be converted to binding antibody units (BAU) according to the First WHO International Standard for anti-SARS-CoV-2 immunoglobulin (27) by multiplication with a factor of 3.2. Semiquantitative measurements of IgA antibodies are expressed as the ratio between the extinction of patient samples and the extinction of a calibrator provided with the kit; values of ≥1.1 are considered as positive IgA antibodies. Surrogate virus neutralizing (sVNT) antibodies were assessed using the cPass SARS-CoV-2 Neutralization Antibody Detection Kit (Nanjing GenScript Biotech Co., Ltd., 211100 Nanjing City, P.R. China) according to the manufacturer’s instructions. This blocking ELISA can detect antibodies that inhibit the interaction between recombinant SARS-CoV-2 receptor-binding domain and angiotensin-converting enzyme 2 in an isotype-independent manner (28, 29). Results are expressed as percent inhibition calculated according to the following formula: % inhibition = [1 − (OD value of sample/OD value of negative control)] × 100; results with ≥30% inhibition were considered positive indicating the presence of SARS-CoV-2 neutralizing antibody.

### Examination of Antigen-Specific Circulating Follicular T-Helper and CXCR5-Negative CD4^+^ Memory T-Cells

**Figure 1:**
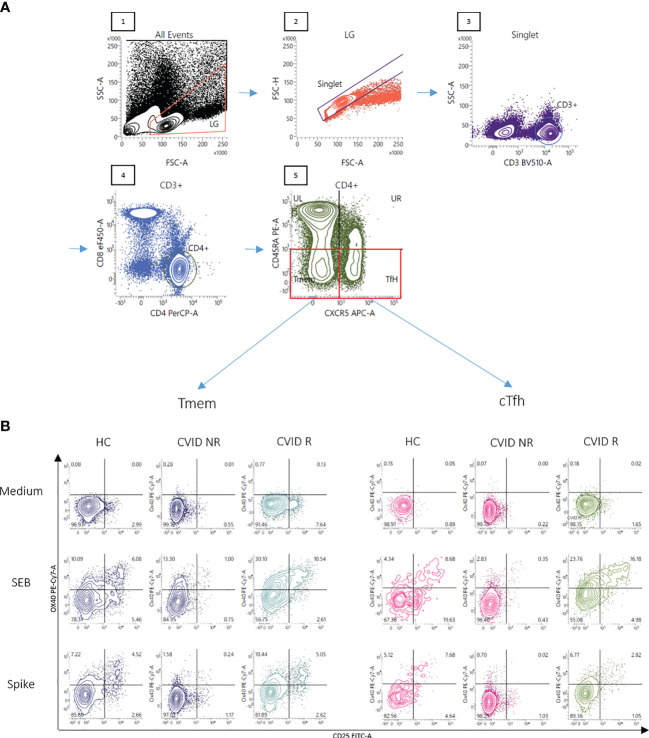
Gating strategy and representative FACS plots of CD4^+^ T memory cells responsive to SARS-CoV-2 spike peptides. Panel **(A)** A lymphocyte gate was applied to a forward - sideward scatter plot of all events (1), followed by doublet exclusion using forward scatter area vs height for cells within the lymphocyte gate (LG) (2). CD3^+^cells were then selected out of singlets (3) and CD3^+^ T-cells were examined for CD4 staining (4). Finally CD3^+^CD4^+^ CD45RA^-^ T-memory cells were divided into CXCR5^-^ (Tmem) and CXCR5^+^ (cTfh) cells (5). OX40(CD134)^+^CD25^+^ cells were finally defined as activated T-cells. Panel **(B)** Activation of Tmem and cTfh cells after stimulation of the cells with SEB or overlapping peptides of SARS-CoV-2 spike protein is shown in a representative healthy control (HC), a CVID patient IgG-responsive to BNT162b2 vaccination (CVID R, SARS-CoV-2 IgG antibody level following the second vaccination more than 33 RE/ml) and a CVID patient IgG-non-responsive following mRNA vaccination (CVID NR, SARS-CoV-2 IgG below 33 RE/ml following the second vaccination). Unstimulated control cells were incubated in medium alone (Medium). The percentages of OX40(CD134)^+^ and CD25^+^ double-positive activated CD4 T cell subsets are shown in the upper right panel of the respective FACS plots.

An activation-induced marker assay was used to detect T cells specific for SARS-CoV2 spike protein (26). Human peripheral blood mononuclear cells (PBMC) were isolated from whole blood by density gradient centrifugation (Lymphoprep; Invitrogen, Lofer, Austria) and cultured in RPMI 1640 medium (GibcoBRL, Invitrogen) containing 2 mM/ml L-glutamine, 100 U/ml penicillin and 100 μg/ml streptomycin (Invitrogen) and supplemented with 10% heat inactivated fetal calf serum (Gibco, Paisley, UK) as previously described (30) at 37°C in the presence of 5% CO2 and 95% humidified air in 24-well tissue culture plates (TC-Platte 24 Well, Standard, F; Sarstedt AG & Co. KG, Nürnbrecht, Germany) in a concentration of 1 × 10^6^ cells/ml and well for two days in the presence of overlapping peptides of immunogenic regions of SARS-CoV2 spike protein (Peptivator SARS-CoV-2 Prot_S, Order.No.: 130-126-701, Miltenyi Biotec) (1 µg of peptides/ml) or the bacterial superantigen staphylococcus enterotoxin B (SEB) (31) in a final concentration of 1 µg/ml. Control cells were incubated in parallel in culture medium only (unstimulated cells). CD4^+^ T-cell subpopulations were then characterized by flow cytometry (32) using commercially available monoclonal antibodies against CD3, CD4, CD45RA, CXCR5 and CXCR3, while upregulation of CD25 and CD134 (OX40) was used to identify activation of CD3^+^/4^+^/45RA^−^/CXCR5^−^ (CXCR5-negative memory T-helper cells, Tmem) and CD3^+^/4^+^/45RA^−^/CXCR5^+^ (circulating follicular T-helper cells, cTfh) cells. Cells were acquired with a FACSVerse (Becton Dickinson; USA) according to the manufacturers recommendations and analyzed using FACSuite software (Becton Dickinson; USA). A lymphocyte and singlet gate was applied, thereby excluding dead cells and cell debris, and at least 100,000 events within this “lymphogate” that were CD3-positive were acquired. The CD3/CD4/CD8/CD45RA/CXCR5 gating strategy used in flow cytometric analysis is depicted in **Figure 1**, panel A. Results are expressed as percent activated (CD134 and CD25 double positive) cells relative to the respective CD4^+^ T cell subpopulation (see **Figure 1** for cell gating strategies and activation marker expression). Alternatively, induction of CD69-expression on activated cTfh and Tmem following stimulation of PBMC with SARS-CoV-2 spike peptides was examined by flow cytometry. Intracellular TNF-alpha expression was examined by flow cytometry after PBMC were stimulated with overlapping peptides of immunogenic regions of SARS-CoV2 spike proteins for two days before brefeldin A was added to block the Golgi-Apparatus and allow for accumulation and detection of intracellular cytokines using a standard protocol. Unstimulated PBMCs were incubated in parallel in medium alone. For measurement of SARS-CoV-2 spike peptide-specific lymphocyte proliferation PBMCs were stimulated for seven days using SARS-CoV2 Spike peptides (1 µg of peptides/ml) before 3H-thymidine was added for 16 h and 3H-thymidine incorporation was assessed as previously described (30). Results are expressed as netto dpm of 3H-thymindine incorporation, calculated by subtracting dpm of unstimulated cell from dpm of stimulated cells.

### Statistical Analysis

Comparison of three or more study groups was performed by calculating the Kruskal–Wallis one-way analysis of variance using Graphpad Prism 6.0.7 software (GraphPad Software, San Diego, CA 92108). Statistical differences between two groups were then confirmed by using the non-parametric two-tailed Mann–Whitney U-test. Values of p <0.05 were considered as statistically significant.

### Data Sharing

Deidentified individual participant data that underlie the reported results will be made available 3 months after publication for a period of 5 years after the publication. Please send all inquiries to hermann.wolf@itk.at.

## Results

### Antibody Responses After BNT162b2 Vaccination Are Impaired in a Subgroup of Patients With CVID

**Figure 2:**
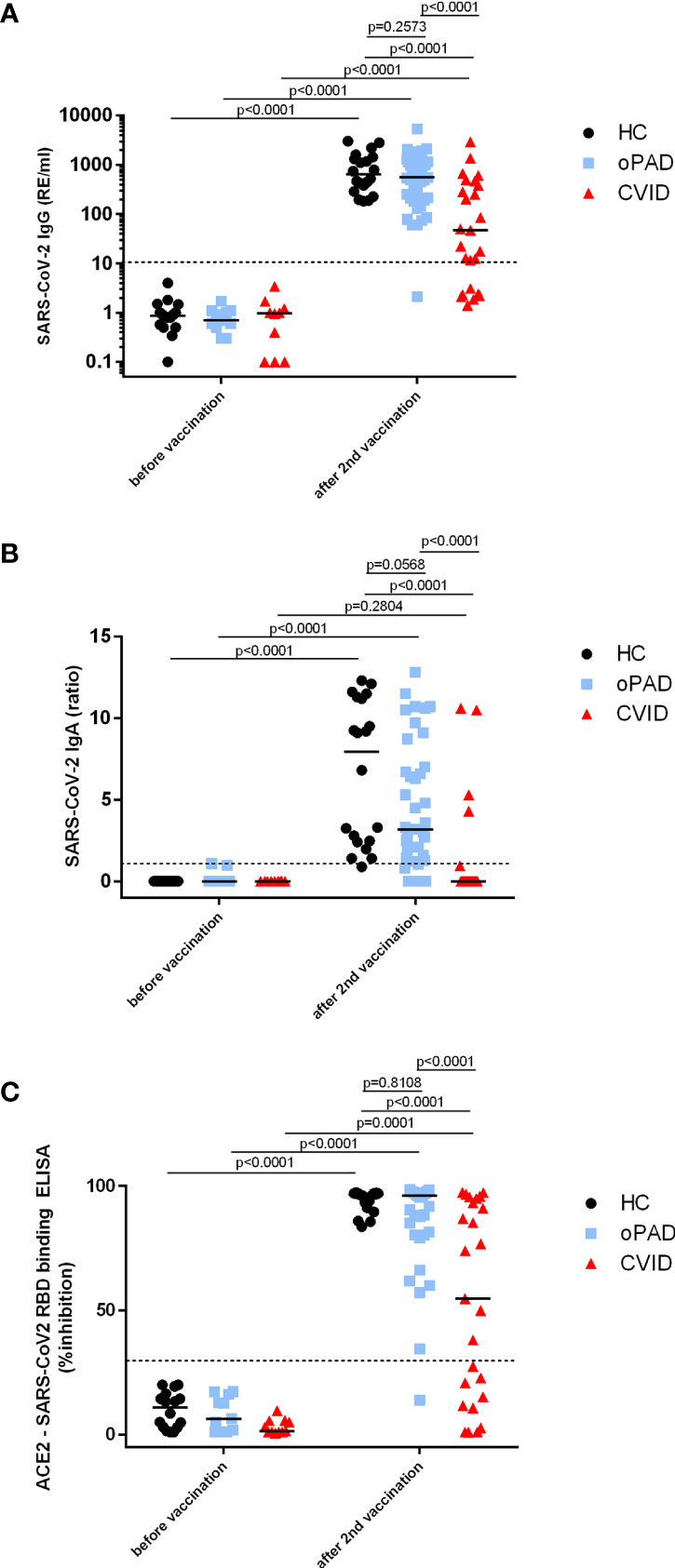
Antibody response following a second BNT162b2 mRNA vaccination in patients with CVID and oPAD. Serum IgG **(A)** and IgA **(B)** antibodies against spike protein of SARS-CoV-2 were examined by ELISA in patients with CVID, patients with milder forms of primary antibody deficiency (other predominantly antibody deficiency, oPAD) and healthy controls (HC) before COVID-19 vaccination and after the second vaccination with the Pfizer-BioNTech COVID-19 vaccine BNT162b2 (Comirnaty). Results for IgG antibodies are expressed as relative units (RE)/ml, the dotted line indicates 11 RE/ml that were used as the cutoff for positivity. Semiquantitative measurements of IgA antibodies are expressed as the ratio between the extinction of patient samples and the extinction of a calibrator provided with the kit; the dotted line indicates a ratio of 1.1 that was considered as the cutoff for positivity. Surrogate virus neutralizing antibodies **(C)** were assessed by testing the ability of serum antibodies (irrespective of isotype) to inhibit the interaction between recombinant SARS-CoV-2 receptor-binding domain and angiotensin-converting enzyme 2 using a blocking ELISA. Results are expressed as percent inhibition; the dotted horizontal line depicts 30% inhibition used as the cutoff for positive SARS-CoV-2 neutralizing antibody. Statistical differences between the two groups depicted in the figure and were determined with a non-parametric two-tailed Mann–Whitney U-test (Kruskal–Wallis H test for all groups: p <0.0001), the median is represented by a horizontal bar.

Healthy controls, oPAD- and CVID-patients had significantly higher SARS-CoV-2 S-protein-specific IgG antibody levels after BNT162b2 vaccination as compared to individuals tested before vaccination (**Figure 2A**). IgG responses in CVID patients as a group however were significantly lower as compared to both healthy controls and oPAD patients (**Figure 2A**); sixteen (51.5%) of 31 CVID patients showed anti-S-IgG antibodies below the detection limit or very low to borderline (below 33 RE/ml, which is three-times the detection limit of 11 RE/ml), while 15 (48.4%) CVID patients produced levels of IgG antibodies comparable to healthy controls (HC) (**Figure 2A**). The IgG antibody level used to separate responders from non-responders was defined following the arbitrary but commonly used rule to define robust, durable and generally more significant IgG antibody levels as those above three-times the detection limit. Among 16 CVID non responders, four patients had IgG antibodies that were low to borderline detectable with levels between 12.5 and 22.5 RE/ml. Only one of these four showed detectable antibodies of 49.9% inhibition in the surrogate neutralization assay. oPAD patients displayed IgG antibody levels after BNT162b2 vaccination that were comparable to healthy controls (**Figure 2A**), and 30/39 oPAD patients (77%) were above the 5% quantile of the healthy control group.

In CVID patients, serum IgA antibodies against spike protein were clearly lower than HC and oPAD, with levels that were comparable to unvaccinated individuals (**Figure 2B**); only four of 31 CVID patients (12.9%) produced detectable anti-S IgA antibodies after two vaccinations (**Figure 2B**). Anti-spike IgA levels were significantly higher in oPAD patients or healthy controls after vaccination as compared to values obtained in unvaccinated individuals, but there was a trend towards lower levels in oPAD patients as compared to healthy controls (IgA anti-spike, median ratio: HC 9.10; oPAD 3.25), and seven oPAD patients failed to produce any IgA antibodies, including two patients with IgA deficiency (**Figure 2B**).

In all three study groups sVNT antibodies were significantly higher after two doses of mRNA COVID-19 vaccination as compared to values detected in vaccine-naïve individuals (**Figure 2C**). Sera from CVID patients that contained significant levels of anti-spike IgG antibodies (>33 RE/ml) also showed significant neutralizing capacity (>30% inhibition) in the sVNT ELISA. In addition, one CVID patient with borderline to low anti-spike IgG antibodies (22.5 RE/ml) showed positive sVNT antibodies (49.9% inhibition), indicating that even moderately low titers of IgG antibodies can have neutralizing activity, at least in the sVNT assay, and one CVID-patient that produced IgA-antibodies (IgA ratio 10.6) but no IgG-antibodies showed inhibitory antibodies in the sVNT assay (76.7% inhibition), indicating that anti-spike IgA antibodies might be responsible for virus neutralization in this patient. In terms of antibody functionality after COVID-19 vaccination patients with oPAD as a group were comparable to healthy controls with five out of 38 patients showing lower levels of neutralizing antibodies as compared to the control group (**Figure 2C**). Only one out of sixteen patients with a selective IgG antibody deficiency against bacterial polysaccharides failed to produce sVNT antibodies (**Figure 2C**) as well as binding IgG and IgA antibodies against spike protein (**Figures 2A, B**).

### Reduced CVID T-Helper Cell Activation After Restimulation With SARS-CoV2 Spike Peptides

**Figure 3:**
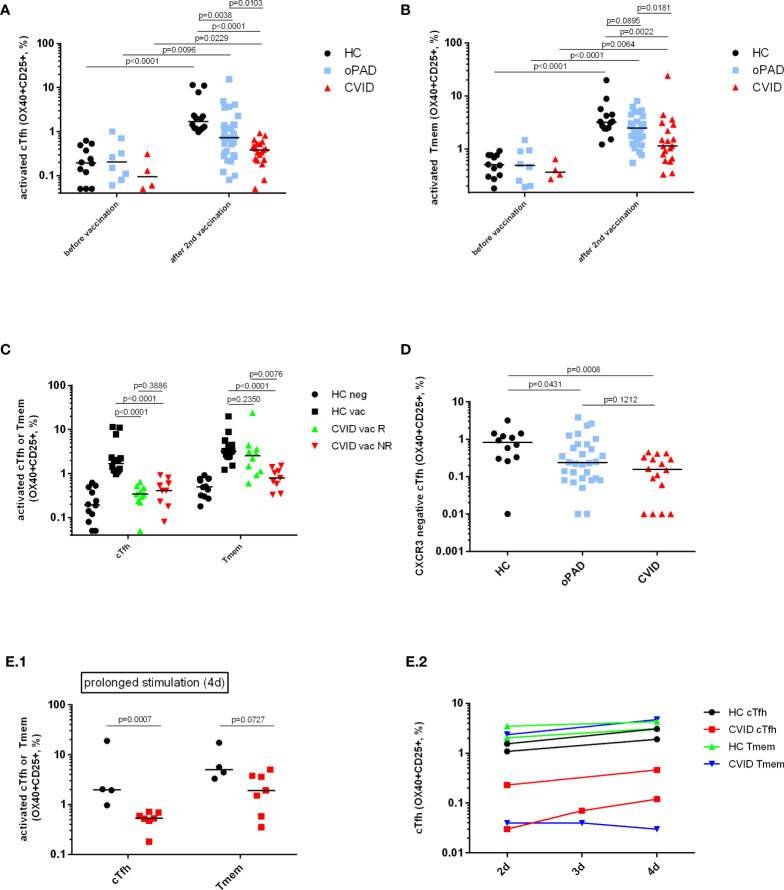
Detection of SARS-CoV-2 spike protein-specific CXCR5^+^ circulating follicular T-helper cells (cTfh), CXCR3-negative cTfh and CXCR5^−^ CD4^+^ T-memory cells (Tmem) by flow cytometry. Human peripheral blood mononuclear cells (PBMC) from healthy controls (HC), CVID patients and patients with other, milder forms of primary antibody deficiency (oPAD), before COVID-19 vaccination and after the second vaccination with BNT162b2, were stimulated for two days using overlapping peptides of immunogenic regions of SARS-CoV2 spike protein (1 µg of peptides/ml). Activation of circulating follicular T-helper cells [CD3^+^CD4^+^CD45RA^-^CXCR5^+^, cTfh, panel **(A)**] and CXCR5-negative CD4 memory T cells [Tmem, panel **(B)**] was determined by measuring upregulation of CD25 and CD134 (OX40) by flow cytometry. Results are expressed as percent CD134 (OX40) and CD25 double positive cells relative to the respective CD4^+^ T cell subpopulation. Unstimulated control cells were incubated in parallel in culture medium only (percent CD134 and CD25 double positive unstimulated cells, median [IQR], before vaccination: HC (n = 12), Tfh 0.04 [0.04], Tmem 0.11 [0.42]; CVID (n = 4), Tfh 0.06 [0.15], Tmem 0.1 [0.2]; oPAD (n = 8), Tfh 0.06 [0.13], Tmem 0.05 [0.01]; after the second vaccination: HC (n = 14), Tfh 0.13 [0.11], Tmem 0.11 [0.42]; CVID (n = 19), Tfh: 0.03 [0.11], Tmem 0.08 [0.14]; oPAD (n = 31), Tfh 0.08 [0.11], Tmem 0.07 [0.53]; Kruskal–Wallis H test: n.s.). Panel **(C)** shows percent of activated, CD134 (OX40) and CD25 double positive cTfh following stimulation of PBMC for two days using overlapping peptides of immunogenic regions of SARS-CoV2 spike protein (1 µg of peptides/ml). PBMC were obtained from healthy controls (HC), CVID responders (anti-spike protein IgG antibody following second vaccination > three times cutoff = 33 RE/ml) and CVID non-responders after the second vaccination against COVID-19. Panel **(D)** shows the percentage of activated CD134 (OX40) and CD25 double positive CXCR3-negative cTfh activated in response to stimulation with SARS-CoV-2 spike peptides. Panel **(E)** shows activation of cTfh and Tmem as assessed by measuring OX40- and CD25-expression following prolonged stimulation with SARS-CoV-2 spike peptides for 4 days [panel **(E.1)**; black circles: healthy controls, red squares:CVID]. In two healthy controls and two CVID patients, cTfh and Tmem activation was examined after two, three and four days of stimulation with SARS-CoV-2 spike peptides [panel **(E.2)**]. Statistical differences between two groups are depicted in the figure and were determined with a non-parametric two-tailed Mann–Whitney U-test (Kruskal–Wallis H test for all groups: p <0.0001), the median is represented by a horizontal bar.

**Figure 4:**
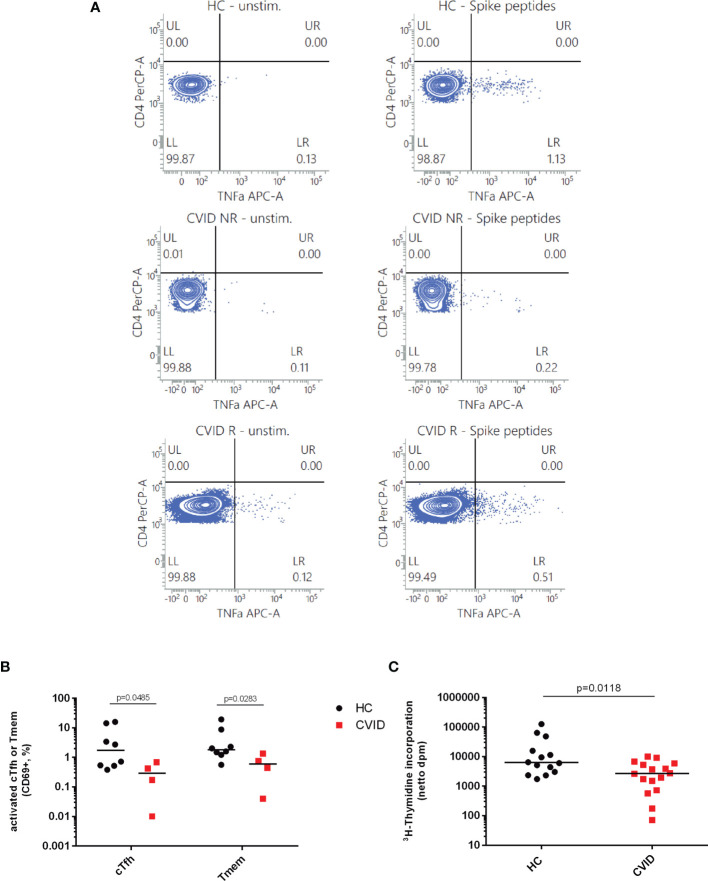
Induction of early activation events such as CD69 expression and TNF-alpha production as well as cell proliferation as a late activation event is reduced in CD4-positive T cells from CVID patients stimulated with SARS-CoV2 spike peptides. TNF-alpha induction was measured as an early activation marker by flow cytometry in CD3^+^CD4^+^CD45RA^−^ cells from CVID patients responding with IgG antibody production following BNT162b2 mRNA COVID-19 vaccination (CVID R), CVID nonresponders (CVID NR) and healthy controls (HC). The figures depicted in panel **(A)** are representative for a total number of four CVID patients and three healthy controls investigated. Unstimulated cells incubated in medium alone were examined in parallel. In panel **(B)** CD69 expression was examined by flow cytometry on cTfh and Tmem of CVID and healthy controls stimulated with SARS-CoV-2 spike peptides; median CD69 expression in unstimulated cells incubated in medium alone was 1.25% cTfh and 0.41% Tmem in healthy controls, and 0.66% cTfh and 0.28% Tmem in CVID patients. In panel **(C)**, PBMCs from healthy controls (HC) and CVID patients were stimulated for seven days using SARS-CoV2 Spike peptides before cell proliferation was examined by measuring ^3^H-thymidine incorporation. Results are expressed as netto dpm of ^3^H-thymindine incorporation, calculated by subtracting dpm of unstimulated cells from dpm of stimulated cells. In unstimulated PBMCs, ^3^H-thymidine incorporation [dpm, median (IQR)] was 932.85 (1,838.28) in healthy controls (n = 14) and 185.15 (463.85) in CVID (n = 16). Statistical differences between two groups were determined with a non-parametric two-tailed Mann–Whitney U-test, the median is represented by a horizontal bar.

PAD patients might benefit from vaccination despite a low or even absent antibody response due to the induction of cellular immunity (24). Activation of both CD4^+^ T cell subsets was significantly reduced in CVID patients as compared to healthy controls but statistically higher as compared to values obtained in CVID patients before vaccination (**Figures 3A, B**). Patients suffering from oPAD displayed significantly higher percentages of activated cTfh cells after vaccination as compared to vaccinated CVID, but values were still significantly lower than in healthy controls (**Figure 3A**). Activation of CXCR3-negative cTfh, a cTfh subset involved in B cell help for IgG antibody production (20), was significantly reduced in CVID patients as compared to healthy controls (**Figure 3D**), indicating that impaired cTfh activation in CVID affected this cTfh subset known to be crucial for B cell IgG antibody production (20). Impaired CVID cTfh or Tmem response to stimulation with the vaccination antigen could also be observed when T cell stimulation was carried out for a prolonged incubation period (**Figures 3E1, E2**), indicating that activation of CD4-positive T cells in CVID is defective rather than delayed. Defective CVID T cell activation in response to spike peptide stimulation could also be observed when antigen-induced production of TNF-alpha, a cytokine produced very early in antigen-specific T cells following TCR-stimulation (33–35) (**Figure 4A**) or antigen-induced CD69 expression, a cell surface antigen expressed by T cells very early after ligation of the TCR/CD3 complex (36) (**Figure 4B**) were examined. Furthermore, antigen-specific T cell proliferation as a relatively late activation event was also reduced in CVID patients (**Figure 4C**).

Antigen-dependent activation of Tmem was significantly higher for healthy adults and oPAD patients after vaccination as compared to values obtained before vaccination (see **Figure 3B**). The median of the CVID group was the lowest of the three groups, significantly lower than in healthy controls or oPAD patients, but still significantly higher as compared to the median of CVID patients before vaccination (**Figure 3B**). Patients with oPAD showed levels of recall-activation of Tmem that were statistically comparable to healthy controls.

### IgG Responsiveness in CVID Patients Following BNT162b2 Vaccination Is Associated With Normal Vaccine-Specific Activation of CXCR5-Negative CD4^+^ Memory T Cells

BNT162b2-vaccinated CVID patients were grouped into anti-spike IgG-responders and non-responders. The threshold for response was determined to be 3 times the positive/negative cut-off (cut-off: 11 RE/ml; threshold responder/non-responder: 33 RE/ml of anti-spike IgG). While both CVID subgroups displayed comparably reduced vaccine-specific cTfh responses, only CVID responders displayed vaccine-specific Tmem activation that was statistically comparable to healthy controls. In contrast, CVID non-responders showed decreased levels of Tmem activation that were comparable to healthy controls tested before vaccination (**Figure 3C**). **Figure 1B** shows representative FACS diagrams showing defective antigen-induced activation of both cTfh and Tmem in a CVID IgG antibody nonresponder, while cTfh activation but not Tmem responsiveness is decreased in the CVID responder.

In addition to normal levels of vaccine antigen-specific Tmem activation, CVID responders had significantly higher levels of serum IgG before immunoglobulin substitution therapy [serum IgG, mg/dl, median (IQR), CVID nonresponders 121 (58), CVID responders 272 (232), p = 0.0116] and a trend towards higher percentages of CD19^+^ lymphocytes [% CD19^+^ lymphocytes, median (IQR), CVID nonresponders 7 (6.9), CVID responders 14 (4), p = 0.070] and higher percentages of MZ-like IgM memory cells among the CD19^+^ B cells [CD27^+^IgD^+^, % of CD19^+^ lymphocytes, median (IQR) CVID nonresponders 8 (11.4)¸CVID responders 23 (19), p = 0.0537] as compared to CVID non-responders (**Table 1**). In contrast there was no difference between CVID responders and non-responders with respect to age at diagnosis, age at time of second vaccination against COVID-19, days between second vaccination and testing of immune response, serum IgA and IgM levels, distribution between the sexes, absolute and relative numbers of CD4^+^ and CD8^+^ cells in peripheral blood, percentage of switched B memory cells among the CD19^+^ lymphocytes, the ability of the patients to produce IgG antibodies against vaccination antigens, and lymphoproliferative responses to PHA as examined by measuring ^3^H-thymidine incorporation (**Table 1**).

### T Helper Cells From CVID Patients Show Defective TCR-Mediated Activation Following Stimulation With *Staphylococcus aureus* Enterotoxin B

**Figure 5:**
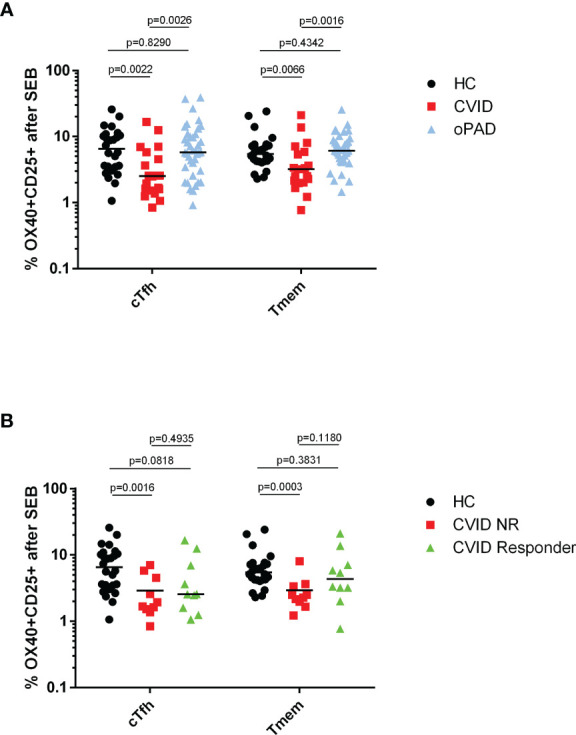
Defective SEB-induced activation of circulating follicular T helper cells and CXCR5-negative CD4 memory T cells from CVID patients. Panel **(A)** human peripheral blood mononuclear cells (PBMC) from healthy controls (HC), CVID patients and patients with other, milder forms of primary antibody deficiency (oPAD) were stimulated for two days with the bacterial superantigen Staphylococcus enterotoxin B (SEB, final concentration 1 µg/ml) before activation of circulating follicular T-helper cells (CD3^+^CD4^+^CD45RA^−^CXCR5^+^, cTfh) and CXCR5-negative CD4 memory T cells (Tmem) was determined by measuring upregulation of CD25 and CD134 (OX40) with flow cytometry. Results are expressed as percent CD134 (OX40) and CD25 double positive cells relative to the respective CD4^+^ T cell subpopulation. Panel **(B)** human peripheral blood mononuclear cells (PBMC) from healthy controls (HC), CVID IgG antibody responders (anti-spike protein IgG antibody following second vaccination > three times cutoff = 33 RE(/ml) and CVID IgG antibody non-responders were stimulated for two days with the bacterial superantigen Staphylococcus enterotoxin B (SEB, final concentration 1 µg/ml) before activation of circulating follicular T-helper cells (CD3^+^CD4^+^CD45RA^−^CXCR5^+^, cTfh) and CXCR5^−^ CD4 memory T cells (Tmem) was determined by measuring upregulation of CD25 and CD134 (OX40) with flow cytometry. Results are expressed as percent CD134 (OX40) and CD25 double positive cells relative to the respective CD4^+^ T cell subpopulation. Statistical differences between two groups given in the figure were determined with a non-parametric two-tailed Mann–Whitney U-test (Kruskal^−^Wallis H test for all groups: p <0.0001), the median is represented by a horizontal bar.

As recall antigen-induced activation of CD4^+^ T cells was defective in CVID patients following the second COVID-19 vaccination—with defective Tmem activation correlated to IgG non-responsiveness—we next investigated whether TCR-mediated activation of CVID CD4^+^ T cells was defective when an antigen-independent stimulus was applied. The results depicted in **Figure 5** indicate that cTfh and Tmem from CVID patients showed significantly reduced levels of activation marker expression in response to SEB stimulation as compared to healthy controls (HC) or oPAD patients, indicating that defective antigen-specific recall activation of cTfh and Tmem following COVID-19 vaccination was associated with a more general defect in TCR-mediated CD4^+^ T cell activation in CVID patients. Levels of SEB-activated CD4^+^ T cell subsets in HC and oPAD were comparable (**Figure 5A**). When we analyzed SEB-induced cTfh and Tmem activation in anti-spike IgG-responsive and non-responsive CVID patients, only the IgG-nonresponders showed significantly reduced SEB-mediated activation of cTfh and Tmem as compared to healthy controls (**Figure 5B**), indicating that impaired anti-spike protein IgG response was associated with defective TCR-mediated CD4^+^ T cell activation.

## Discussion

The present study shows that overall 76% of patients with primary antibody deficiency produced protective anti-SARS-CoV-2 IgG antibodies following two doses of the mRNA vaccine BNT162b2, supporting the recommendation that patients with primary antibody deficiency should be vaccinated against COVID-19, preferentially using an mRNA vaccine (https://www.cdc.gov/vaccines/covid-19/clinical-considerations/covid-19-vaccines-us.html). In the different forms of primary antibody deficiency the likelihood of a positive IgG response depends on the severity of the immunological phenotype. Patients with immunoglobulin isotype deficiency and/or selective IgG antibody deficiency against polysaccharide antigens had a largely normal capacity to produce neutralizing antibodies after two vaccinations with BNT162b2 (38/39 = 97.4% were IgG responders). In contrast, patients with CVID have in common a defective IgG production to various antigens, as evaluated by repeated serum immunoglobulin measurements, B-cell enumeration and phenotyping, and diagnostic vaccination (10, 37, 38). Accordingly, more than half of our CVID cohort failed to mount a significant IgG response following BNT162b2 vaccination. Along these lines it is remarkable that in our study as well as in previous reports (18, 19) a significant subgroup of patients with CVID vaccinated with BNT162b2 against SARS-CoV-2 produced vaccine-specific IgG antibodies with levels comparable to healthy controls. The immunoglobulin products our patients received at the time of testing were negative for SARS-CoV-2 spike protein-specific IgG antibodies, and IgG-antibodies against SARS-CoV-2 nucleocapsid protein were negative in all vaccinated patients (data not shown), indicating that the observed anti-spike IgG responses were indeed produced by the patients in response to mRNA vaccination and not passively transferred by ongoing immunoglobulin substitution therapy. While the IgG responses of the CVID patients against other antigens were not reported in the two previous studies (18, 19) it is interesting to note that SARS-CoV-2 IgG responders and non-responders among our CVID patients had comparably defective IgG antibody responses to a variety of other microbial pathogens and vaccination antigens such as staphylococcal or streptococcal toxins, viral antigens such as measles, mumps, rubella or VZV, pneumococcal or Hib capsular polysaccharides, and tetanus or diphtheria toxoids, indicating a potentially increased immunogenicity of mRNA vaccines in CVID patients, thus stimulating IgG antibodies when more conventional forms of antigen delivery failed. IgG antibody responses after vaccination with influenza virus antigen, bacterial polysaccharides or diphtheria and tetanus toxoids in selected CVID patients have been previously associated with milder clinical symptomatology (39), higher levels of switched B memory cells (40) or more circulating plasmablasts (41) as compared to nonresponders. In our study CVID responders showed significantly higher levels of serum IgG before immunoglobulin substitution therapy, and a trend towards higher percentages of CD19^+^ lymphocytes and higher percentages of MZ-like IgM memory B cells as compared to CVID non-responders, indicating that together with new mRNA vaccine technology less severe impairment of immunity might be responsible for intact IgG responses to BNT162b2 vaccination in these patients.

Most CVID patients also have IgA deficiency (9) and IgA deficiency was recently postulated as a risk factor for developing severe COVID-19 in CVID patients (42). The overall majority (87%) of our CVID patients failed to produce IgA antibodies against SARS-CoV-2 spike protein, including even patients who were capable of producing IgG antibodies following BNT162b2 vaccination. In contrast, the IgA response after BNT162b2 vaccination was comparable in patients with milder forms of primary antibody deficiency and healthy controls (**Figure 2B**). Impaired IgA response following vaccination has been described as a prognostic marker in CVID (43), but whether deficient IgA responses in immunized CVID patients are relevant for protection against SARS-CoV-2 infection remains to be determined.

Although the mechanisms leading to BNT162b2 vaccine responsiveness in PAD patients are likely diverse, our results show that a preserved antigen-specific CD4^+^ T memory cell response might play an essential role. CXCR5-positive Tfh cells are known to be important for the formation of germinal centers, B cell proliferation, antibody diversification and affinity maturation, isotype switching and the differentiation of B cells into memory cells and antibody-secreting plasma cells (20, 23), and antibody nonresponsiveness to H1N1 influenza virus vaccine has been correlated with altered vaccine-specific Tfh responses in immunocompromised populations (21). In our study impaired activation of CXCR5-negative CD4^+^ memory cells but not cTfh characterized IgG nonresponsiveness in patients with CVID, while antigen-specific activation of CXCR5-positive cTfh was comparably reduced in CVID BNT162b2-IgG-responders and nonresponders. The mechanism whereby CXCR5-negative CD4^−^ T cells might influence B cell responses remains to be determined. Of particular interest in this respect are the recently described CXCR5-negative human peripheral helper T cells which have first been shown to expand in autoimmune diseases (44). Up to now participation of CXCR5-negative peripheral helper T cells in B cell responses has been limited to autoantibody and alloantibody production (44, 45), but these helper T cells could also play a role in mRNA vaccine-specific antibody responses. Both T helper subsets exert B helper activities using comparable mechanisms to some extent, e.g., *via* IL-21 expression (46) and baseline levels of CXCR5-negative peripheral helper T cells strongly predict the ability to produce anti-spike RBD IgG antibodies in immunocompromised transplant patients vaccinated against COVID-19 (47). Further studies are needed to confirm that CXCR5-negative peripheral helper T cells induce B cell responses in CVID patients following COVID-19 vaccination, but it is intriguing to note that peripheral T helper cells have been implicated in extrafollicular B cell differentiation (46), and IgG responses in CVID patients vaccinated against COVID-19 have been ascribed to atypical memory B cell responses producing low affinity spike protein-specific antibodies as a result of extrafollicular B cell differentiation (18). The emergence of extrafollicular B cell responses during symptomatic COVID-19 has been recently described, thus potentially disrupting the normal follicular B cell differentiation known to result in long-term protection (48). Although our results suggest that the IgG antibodies produced by CVID responders with the help of “atypical” CD4 helper cells are functional shortly after vaccination, it remains to be determined whether CVID responders mount a booster response following subsequent vaccination that is required for long-term protection, in particular against new variants of SARS-CoV-2.

Our study shows that activation of cTfh in response to stimulation with a vaccination antigen is defective in CVID, and that prolonged stimulation with the antigen does not restore T cell activation, indicating that CD4 T cell activation is not merely delayed in CVID (**Figure 3E**). Abnormalities in CD4^+^ T cell activation could be responsible for impaired antigen-specific CD4^+^ memory cell activation leading to IgG non-responsiveness, as TCR-mediated activation of CD4^+^ T cells after SEB stimulation was defective in CVID BNT162b2 IgG nonresponders. Defective TCR-mediated signalling has been previously described in CVID T cells (30) and could play a role in IgG non-responsiveness, as inhibition of TCR-mediated Tfh cell activation has been shown to impair antibody responses and T cell help for immunoglobulin production *in vitro* (49). Defective TCR-mediated T cell activation in CVID could affect Tfh more severely than other types of CD4-positive T cells such as Tmem. Along these lines it has been described that activation of Tfh requires particularly strong and sustained TCR/ligand interactions (50), activation requirements that could be affected by the costimulation defect previously shown to lead to defective TCR-dependent T cell activation in CVID (30).

A major limitation of our study is that we examined vaccination responses at one time point only, as delayed responses to SARS-CoV-2 vaccination have been reported in immunocompromised populations (51, 52). Longevity and functionality of B cell responses after vaccination depend on intact CD4^+^ T cell responses, in particular of the Tfh subset (23). It remains to be determined whether decreased Tfh responses in PAD as compared to healthy controls lead to a more rapid decline of IgG antibody titers and/or impaired booster responses. Alternatively additional booster immunizations could lead to enhancement of SARS-CoV-2 cTfh and antibody responses, as intact Tfh responses have been reported in CVID following vaccination against influenza, an antigen repeatedly encountered in adult life (16).

The anti-spike IgG antibodies produced by PAD patients in our study were functional in a surrogate virus neutralization test, as has also been reported previously (19), which is in contrast to the previous observation that memory B cells with high specificity against the receptor binding domain of the viral spike protein, cells known to produce most of the neutralizing antibodies against SARS-CoV-2, are undetectable in COVID-19 vaccinated CVID patients (18). The diagnosis of CVID is made by exclusion in at least 90% of the patients, which leads to the considerable immunological, clinical and genetic heterogeneity of patients that might explain some of the discrepancies between our results and previous studies (18, 19). Our findings are nevertheless very encouraging with respect to the question whether patients with PAD should be vaccinated against COVID as even a subgroup of CVID patients that were deficient in IgG responses against various other antigens produced significant levels of functional anti-spike IgG antibodies that should confer protection against infection with SARS-CoV-2 to this vulnerable population.

## Data Availability Statement

The original contributions presented in the study are publicly available. This data can be found here: https://www.ncbi.nlm.nih.gov/snp/ accession numbers rs1131690788 and rs1724867456.

## Ethics Statement

The study was conducted in accordance with the Declaration of Helsinki and fulfils the guidelines of the Austrian Agency of Research Integrity (OeAWI). With respect to the clinical immunological analyses this study was approved by the Ethics Committee of the Immunology Outpatient Clinic as a study using the biobank of residual specimen of the Immunology Outpatient Clinic. According to the Ethics Committee of the City of Vienna and the legal regulations to be applied (§15a Abs. 3a Wiener Krankenanstaltengesetz) no additional ethics committee evaluation is required for a non-interventional study using data collected as part of the routine medical care the patients received. The patients gave their informed consent that anonymized data collected as part of the routine medical attendance (serum antibody and flow cytometry analysis, activation-induced marker assay) could be included in a scientific publication. All patient information in this study is anonymized and deidentified. No extra intervention was carried out.

## Author Contributions

KS, CG, and RS performed the T cell activation assays and interpreted the respective results. HA performed the antibody measurements. KS created the figures and tables. KS and HW interpreted and analyzed the overall results, took overall responsibility for the research performed in this study and actively wrote the manuscript. PI, JW, CG, and HW cared for the patients and provided samples. Critical revision of the article was done by ME, CG, and MF. All authors listed have made a substantial, direct, and intellectual contribution to the work and approved it for publication.

## Funding

This study was supported by the Österreichische Forschungsförderungsgesellschaft mbH (grant number 881639), and by the Jeffrey Modell Foundation, the Johns Hopkins Research Foundation and the Robert A. Good Endowment.

## Conflict of Interest

Authors KS and ME were employed by the company Biomedizinische Forschung & Bio-Produkte AG that had no role in the design of this study or during its execution, analyses, interpretation of the data and decision to submit the present manuscript.

The remaining authors declare that the research was conducted in the absence of any commercial or financial relationships that could be construed as a potential conflict of interest.

## Publisher’s Note

All claims expressed in this article are solely those of the authors and do not necessarily represent those of their affiliated organizations, or those of the publisher, the editors and the reviewers. Any product that may be evaluated in this article, or claim that may be made by its manufacturer, is not guaranteed or endorsed by the publisher.

## Abbreviations

CVID, common variable immunodeficiency; HC, healthy controls; oPAD, milder forms of primary antibody deficiency other than CVID, e.g. immunoglobulin isotype deficiency or selective antibody deficiency; SEB, Staphylococcal enterotoxin B; cTfh, circulating T follicular helper cell; IQR, interquartile range.
